# High performance multi-purpose nanostructured thin films by inkjet printing: Au micro-electrodes and SERS substrates†

**DOI:** 10.1039/d2na00917j

**Published:** 2023-02-16

**Authors:** Simona Ricci, Marco Buonomo, Stefano Casalini, Sara Bonacchi, Moreno Meneghetti, Lucio Litti

**Affiliations:** a Department of Chemical Sciences, University of Padova Via Marzolo, 1, 35131 Padova Italy lucio.litti@unipd.it +39-049-8275530; b Department of Informatic Engineering, University of Padova Via Gradenigo 6/b 35131 Padova Italy

## Abstract

Nanostructured thin metal films are exploited in a wide range of applications, spanning from electrical to optical transducers and sensors. Inkjet printing has become a compliant technique for sustainable, solution-processed, and cost-effective thin films fabrication. Inspired by the principles of green chemistry, here we show two novel formulations of Au nanoparticle-based inks for manufacturing nanostructured and conductive thin films by using inkjet printing. This approach showed the feasibility to minimize the use of two limiting factors, namely stabilizers and sintering. The extensive morphological and structural characterization provides pieces of evidence about how the nanotextures lead to high electrical and optical performances. Our conductive films (sheet resistance equal to 10.8 ± 4.1 Ω per square) are a few hundred nanometres thick and feature remarkable optical properties in terms of SERS activity with enhancement factors as high as 10^7^ averaged on the mm^2^ scale. Our proof-of-concept succeeded in simultaneously combining electrochemistry and SERS by means of real-time tracking of the specific signal of mercaptobenzoic acid cast on our nanostructured electrode.

## Abstract 2

Excellent performances in SERS and electronics are obtained for a series of replicates of Au nanoparticle inkjet-printed nanostructured substrates.

## Graphical abstract

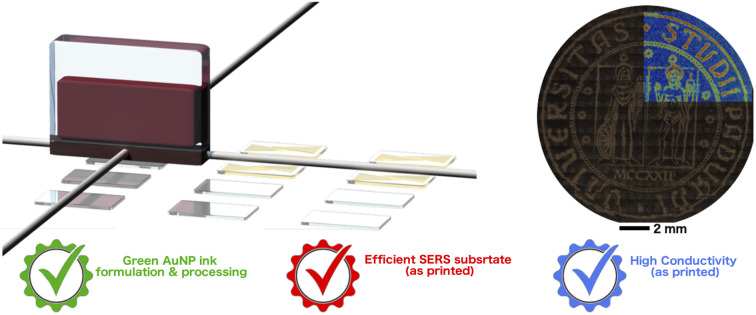

## Introduction

Nowadays, smart surfaces based on innovative thin films are ubiquitous, spanning from standard electronic devices to the most ground-breaking developments on transistors,^1,2^ energy harvesting,^3,4^ sensors,^5^ and catalysts.^6–8^ Nonetheless, the majority of promising lab-scaled technologies have often encountered difficulties in attracting industrial investments, due to their poor compliance with a high throughput. For instance, lithography is a benchmark in terms of manufacturing processing, but it relies on the use of hazardous and/or expensive materials, such as photoresists, which cannot be defined as environment friendly. Furthermore, lithography requires intrinsically relevant expenses due to the extensive use of the clean room's facilities and highly specialized personnel. Another manufacturing benchmark consists of the spin-coating technique, which is in contrast characterized by high material losses. Other techniques such as deep-coating or screen-printing are more efficient than spin-coating, as far as the liquid ink may be recycled and reused to a certain extent.^9^ Within this context, inkjet printing is certainly one of the most promising techniques, due to its easy scalability, cost-effectiveness, user-friendliness, reduced material waste, sustainability, and high versatility towards substrates (*viz.* from silicon to plastics and paper).^10^ This technique is rather versatile, and hence, a droplet can be cast by continuous jetting (CIJ) or by the drop-on-demand (DOD) strategy onto any type of substrate.^11,12^ As a result, it is exploited for the preparation of devices spanning from organic thin-film transistors (OTFTs)^2,13,14^ and organic light-emitting diodes (OLEDs)^15,16^ to solar cells.^17,18^ Noteworthy, the spreading of metal nanoparticle-based inks has boosted the preparation of all-inkjet printed devices, allowing a significant cost reduction and an improvement in the sustainability of the overall process. To the best of our knowledge, the majority of the publications rely on commercial gold and silver inks containing extra additives, such as silicone and glycerol, which are essential for improving colloidal stability.^4,19,20^ Concerning thin-film electrodes, thermal post-treatment is usually mandatory to enable and/or enhance electrical conductivity because it induces additive degradation and nanoparticle fusion. Although these beneficial effects are fundamental for achieving high-performance electrodes, it is a clear advantage to avoid this further step enabling a more straightforward and cost-effective procedure in terms of power consumption, thus complying with the so-called additive manufacturing. Furthermore, high temperatures can severely damage some technologically relevant components such as organic semiconductors and/or dielectric materials, hence limiting severely the portfolio of materials compliant with ink-jet printing. As a consequence, the achievement of the entire manufacturing based on inkjet printing will be an added value for the corresponding nanoparticle technology. In this regard, Minari *et al.* developed a strategy to achieve gold conductivity at room temperature, by adding aromatic molecules as stabilizers of the gold nanoparticles.^21^ It represents an excellent example of environment-friendly manufacturing, but it cannot be applied, for instance, in sensing applications, as the gold surface is passivated. Among the inkjet-printed thin films for optical purposes, the ones used for surface enhanced Raman scattering (SERS) deserve particular attention. For instance, active Raman molecules can be easily detected once adsorbed onto nanostructured metal surfaces due to the intrinsic SERS amplification. This stems from the combination of an electromagnetic enhancement due to the excitation of the surface plasmons promoted by the substrate, and a chemical enhancement mainly due to charge transfers between the molecules and the substrate.^22^ Apart from the surface nanostructuration, the absence of impurities is, therefore, a fundamental property of this technique, as far as the SERS effect vanishes just a few nanometres away from the metal surface. SERS is becoming a renowned technique in sensing applications, thereby providing a vibrational spectrum featuring relevant chemical information,^23^ bright as fluorescence, compatible with water-soluble samples and suitable for quantitative estimations.^24,25^ Notwithstanding this, the preparation of SERS-active substrates with high reproducibility and uniformity is still an open challenge, especially when solution-processed technologies are employed.^26^ Moreover, the highest enhancement factors (EF) achieved by SERS substrates printed on paper or glass, when reported, are in the order of 10^4^–10^8^.^27–30^ To the best of our knowledge, no inkjet-printed substrates have been reported to comply with both optical and electrical applications yet.

Herein, we demonstrate how the inkjet printing technique can be optimized to obtain gold nanostructured electrodes. In particular, we conceived two novel and sustainable inks. Our smart substrates showed promising features; in fact, SERS and electrical read-outs have been successfully demonstrated. Advanced morphological and structural characterization studies validated the unprecedented properties of these nanotextured surfaces, namely as high-performance, multi-purpose, environment-friendly and sustainable substrates. These thin films were finally used for electrochemical-SERS experiments on mercaptobenzoic acid (MBA) for proving the practical applicability of our multifunctional SERS-active microelectrodes, thereby revealing clear information associated with the surface chemistry of MBA.

## Results and discussion

### Design and manufacturing of the printed thin films

**Fig. 1:**
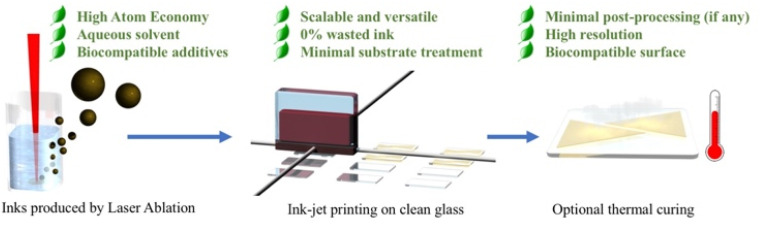
A schematic sketch of the stepwise processing of AuNPs. Every step shows its green chemistry principles.

The nanostructured SERS active electrodes were designed according to the green chemistry principles, namely involving water-based formulations, high atom-economic processes, and no usage of hazardous materials. The overall workflow is schematized in Fig. 1: (i) gold nanoparticle (AuNP) synthesis by laser ablation and ink formulation, (ii) inkjet printing optimization, and (iii) optional thermal sintering. Laser ablation is a top-down technique to produce nanoparticles either in aqueous or organic solutions without any capping agents or stabilizers.^31–33^ Although these are excellent features, the use of additives is still necessary to enhance the colloidal stability along the inkjet printing process. Specifically, l-cysteine and polyvinyl alcohol (PVA) are both sustainable, non-toxic, water-soluble and already used as colloidal stabilizers.^34–38^l-Cysteine is an amino acid bearing a thiol moiety, which is widely known to form strong covalent bonds with Au surfaces. Its charge can be easily tuned to achieve a sufficient number of negative charges to preserve colloidal suspension from aggregation, especially when it undergoes relevant pressure changes into the cartridge. PVA is a biodegradable and biocompatible polymer, which is also needed to increase ink viscosity. According to the chemical features of these two stabilizers, two formulations have been conceived and explored. On one hand, the laser-ablated AuNPs were capped with a sub-monolayer amount of cysteine (see the ESI† for more details) and, on the other hand, 1 g L^−1^ of PVA was added to the laser-ablated AuNPs. Hereafter, they are denoted as AuNP_Cys and AuNP_PVA, respectively (see the ESI† for more details). UV spectroscopy and TEM imaging allowed the appreciation of AuNP_Cys and AuNP_PVA with respect to the pristine Au NPs (Fig. S1†), both characterized by a plasmon peak at 522 nm and a size distribution centred at 16 nm.

A rectangular layout (*i.e.* nominal area equal to 2 × 10 mm^2^) was selected as the pattern to be inkjet-printed. Therefore, 12 identical inkjet-printed thin films have been printed on glass substrates, 6 for each type of ink formulation. As already mentioned, thermal sintering is a general fundamental approach for printed nanostructured electrodes to increase their electrical conductivity due to NP fusion.^39–41^ However, high sintering temperatures can narrow down the portfolio of materials to be used as substrates, especially flexible ones.^21^ To gain insights into the effect of sintering on our films, their electrical optical properties were investigated with and without the thermal treatment. In particular, our systematic investigation relies on the comparison of the three sintered samples (*T*_sintering_ = 500 °C for 3 hours under an air atmosphere) with respect to the other three pristine samples. The sintering conditions were defined to allow the complete degradation of both PVA^42^ and l-cysteine.^43,44^

### Morphological characterization of the printed thin films

**Fig. 2:**
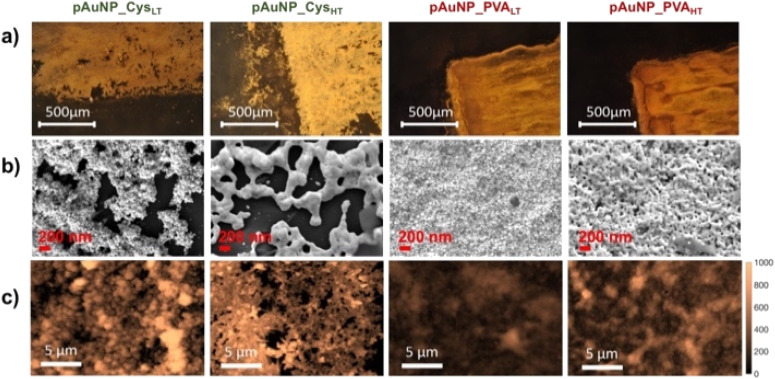
(a) Dark-field optical microscopy images with a 5× objective, (b) SEM images, and (c) AFM topography images. The colorbar at the right applies to all images and refers to the elevation in nanometers.

The morphology is one of the most important figures of merits related to an optoelectronic thin film. For sake of clarity, pAuNP_Cys_LT_, pAuNP_Cys_HT_, pAuNP_PVA_LT_ and pAuNP_PVA_HT_ stand for the printed nanostructured thin film based on AuNPs either at low temperature (LT), *i.e.* no sintering, or high temperature (HT), *i.e. T*_sintering_ = 500 °C, having cysteine and polyvinyl alcohol, respectively. Fig. 2a shows that pAuNP_PVA samples have more defined edges than pAuNP_Cys. The combination of optical microscopy, scanning electron microscopy (SEM) and atomic force microscopy (AFM) highlights that pAuNP_PVA features a higher degree of homogeneity than pAuNP_Cys from the very first printed layer (Fig. S2–S7†). This higher degree of homogeneity can be justified considering the blending effect of the PVA polymer, which improves the printability of our water-based ink.^45–47^ In contrast, l-cysteine solution is not reported to influence the solvent viscosity or other rheological properties. Dark-field and SEM images also show more compact layers for the pAuNP_PVA samples at higher magnification (Fig. S4 and S6†), highlighting the significant role of PVA in the final manufactured electrode. SEM analysis allows a deeper examination of the nanostructuration to be achieved (Fig. 2b and S2–S4†), which confirms a more homogenous deposition for the pAuNP_PVA samples. Furthermore, it was possible to evaluate the impact of sintering related to the differences between the colloidal stabilizers. Both pAuNP_Cys_LT_ and pAuNP_PVA_LT_ present a nanostructured surface, in which the AuNPs are perceivable. However, as a result of the sintering step, pAuNP_Cys_HT_ clearly shows extensive AuNP aggregates, whereas pAuNP_PVA_HT_ shows a homogenous nanostructured morphology. Both outcomes are likely due to AuNP melting.

AFM imaging enables a detailed evaluation of surface roughness. In fact, pAuNP_Cys_LT_ substrates exhibit a roughness of 0.136(±0.017) μm, which slightly decreases after the sintering process to 0.130(±0.06) μm (Fig. 2c and S2, S3, S7 and S8†), whereas pAuNP_PVA_LT_ ones show two different regions characterized by different roughness values, namely 0.047(±0.012) μm and 0.103(±0.047) μm, respectively. These areas can be associated with the brighter and darker ones observed by optical microscopy operated in dark-field mode. In other terms, the darker areas correspond to the rougher surfaces, while the brighter areas are the smoother surfaces (Fig. S7†). The arrangement of these two areas can be ascribed to the confinement of a larger quantity of PVA into the smoother (*viz.* brighter areas) areas, which appear less resolved by AFM. This interpretation is also corroborated by μ-Raman spectroscopy (Fig. S9†), revealing PVA characteristic bands in these regions. Lastly, their thicknesses were found to be about 300 nm for the pAuNP_Cys samples and 250 nm for the pAuNP_PVA ones (Fig. S8†).

**Fig. 3:**
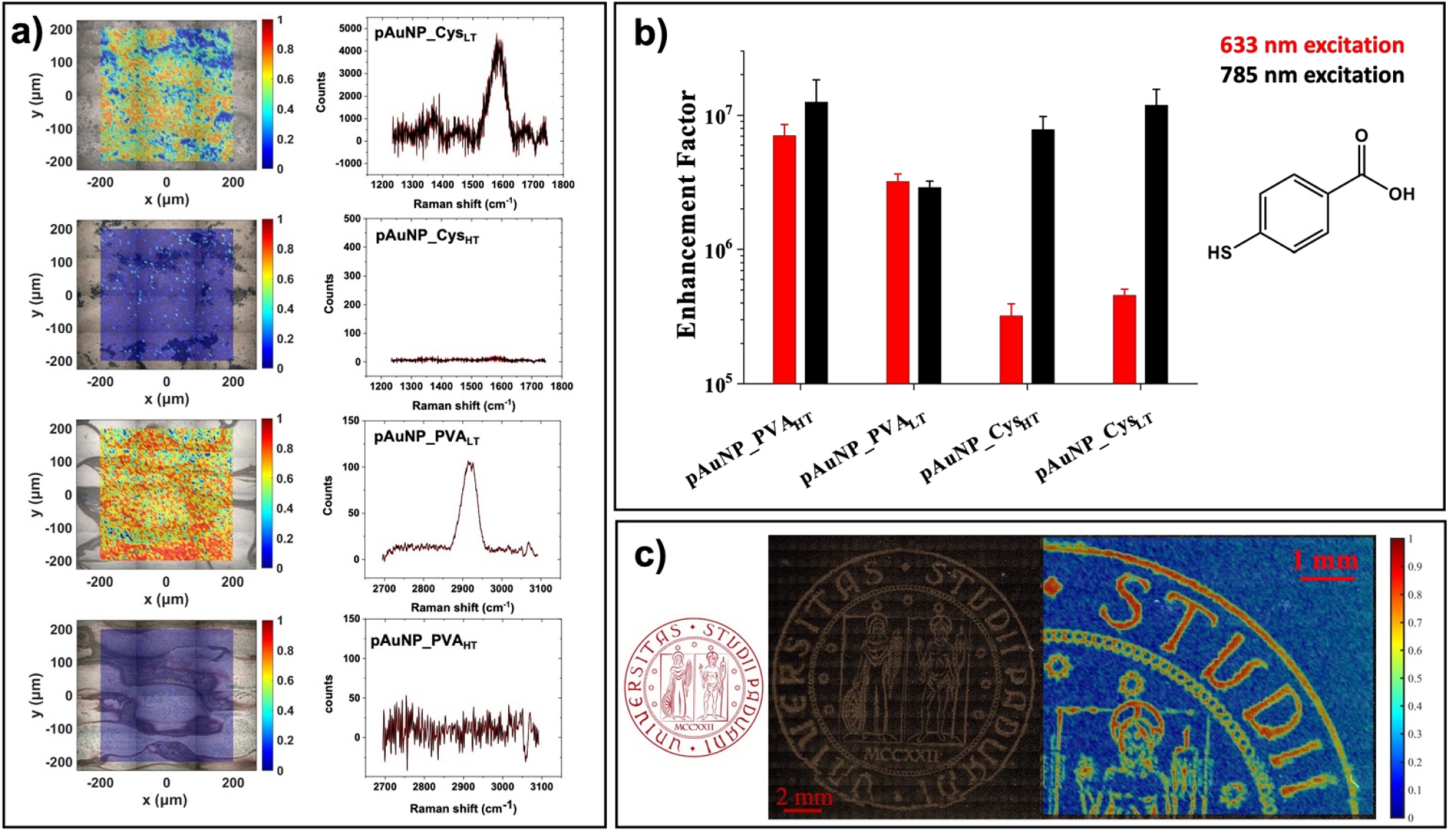
(a) μ-Raman maps (633 nm excitation and 50× objective). (b) The EF was calculated for all replicates and averaged, at 633 nm excitation (red symbol) and 785 nm excitation (black symbols); the error bars represent the standard errors. (c) Padova University logo printed with the AuNP_PVA ink formulation with only one layer and any thermal curing. The colourmap represents Pearson's correlation coefficient with the PVA spectrum.

As previously mentioned, the sintering of cast AuNPs is a standard procedure to make the thin films more efficiently conductive. Aiming at deeply investigating the partial presence of the colloidal stabilizers in the sintered thin film, Raman spectroscopy (Fig. 3a) and energy dispersive X-ray (EDX) analysis (Fig. S10†) were carried out. Extensive statistical analysis (6400 spectra) was carried out in an area equal to 0.4 × 0.4 mm^2^, at a resolution of 5 μm. These spectra were compared with the Raman spectra of pristine AuNP_Cys and AuNP_PVA inks (Fig. S11†). The most characteristic signals of the two additives are recognized as the peak at 1580 cm^−1^, assigned to the amine bending for l-cysteine,^48^ and the peak centred at 2915 cm^−1^, corresponding to the CH stretching for PVA.^49^ Pearson's correlation coefficients were calculated to account for the correspondence between the spectra from the μ-Raman maps and the relative pristine inks. A value higher than 0.6 is considered a clear indication of a satisfying positive comparison with ref. 24 The μ-Raman maps confirmed the complete degradation of both colloidal stabilizers. These data are coherent with the proof collected by EDX analysis (Fig. S10†).

### SERS characterization

Surface homogeneity and nanostructuration are considered demanding challenges for the production of SERS substrates by solution-processing methods.^50^ SERS active substrates rely on the presence of the hot-spots, which are localized regions of intense local field enhancement which play a key role in yielding the output signals.^51^ As a result, it is not trivial to achieve a satisfactory amount of bright hot-spots on large-area SERS substrates. Herein, the SERS efficiencies of our printed thin films were evaluated by means of their enhancement factors (EFs) mediated on a macro area, namely on the scale of mm^2^. Specifically, more than 1500 spectra were acquired over a surface as large as 1.5 × 0.4 mm^2^, at a resolution of 20 μm, thus evaluating both the uniformity of the substrates (intra-sample over a large surface) and the reproducibility (inter-sample through three replicates) of the results. The EF was calculated according to the formalism of the so-called average SERS enhancement factor,^52^ in which the SERS signal and the Raman signal of the same band of a molecule are weighted by the number of molecules probed and compared (see the ESI† for more details). The characteristic and intense peak of 4-mercaptobenzoic acid (4-MBA), at around 1580 cm^−1^, corresponding to the C=C symmetric stretching vibration, acted as a SERS probe (Fig. S12†).^23–25,52,53^ 4-MBA allowed an accurate comparison between the Raman and SERS signals and therefore, SERS EF calculations (Fig. S13 and S14†). Fig. 3b shows the EF values for both laser lines: 633 nm (red symbols) and 785 nm (black symbol) for the three replicates averaged. According to the EF values related to pAuNP_Cys_LT_ and pAuNP_Cys_H_T__ with 633 nm excitation, these values are rather close, namely 4.6(±0.5) × 10^5^ and 3.2(±0.7) × 10^5^, respectively. A similar behaviour is observed for pAuNP_PVALT and pAuNP_PVAHT at the same laser line, namely 3.2(±0.4) × 10^6^ and 7.0(±1.5) × 10^6^. Although there are discrepancies in the morphologies of these 4 SERS active substrates, the sintering process does not induce a clear improvement of the SERS signal. Concerning the measurements with the 785 nm laser line, a similar trend is observed, namely, the sintering step is not yielding a clear advantage in the SERS performance. Summarising, pAuNP_Cys_LT_, pAuNP_Cys_HT_, pAuNP_PVA_LT_ and pAuNP_PVA_HT_ show the following EF values: 1.2(±0.4) × 10^7^, 7.8(±2.0) × 10^6^, 2.9(±0.3) × 10^6^ and 1.3(±0.6) × 10^7^, respectively. It highlights how robust and efficient are these nanostructured thin films in respect of the SERS EF, as they perform similarly like as as-printed or after thermal sintering. If one compares pAuNP_Cys_LT_ and pAuNP_PVA_LT_, it is clear that the former substrate has a superior performance likely ascribed to a more efficient resonance with 785 nm excitation, as previously documented.^31^ Moreover, our methodology has been further challenged to define which spatial resolution can be achieved. For this purpose, the logo of the University of Padova has been selected. This pattern has been manufactured on a glass substrate with the AuNP_PVA ink (see Fig. 3c). This proof of concept allowed us to define a spatial resolution down to the microscale within a pattern featuring an area equal to 14 × 14 mm^2^. This promising result, coupled with the limited amount of ink necessary, will be of great interest in large-area and environment-friendly electronics.

### Electrical characterization

Since morphology and SERS features were successfully characterized, we explored the possibility to use them as printed electrodes. First, the sheet resistance was measured by using a four-probe system, showing a remarkable value of 10.8(±4.1) Ω per square and 7.9(±8.7) Ω per square for pAuNP_Cys_LT_ and pAuNP_Cys_HT_, respectively. Although SEM and AFM highlighted differences in the morphology of these samples, their conductivity remains unaltered within the error bars. Our rationale relies on the key role played by l-cysteine, which, on one hand allows the stabilization of the ink and, on the other hand, acts as a molecular bridge between the AuNPs, despite the limited amount added to the ink solution. More importantly, pAuNP_Cys_LT_ outperforms electrodes made of graphite and carbon black with similar thicknesses (Fig. S16†).^54,55^ Regarding pAuNP_PVA samples, two relevant differences with respect to pAuNP_Cys_LT_ and pAuNP_Cys_HT_ appear: (i) pAuNP_PVA_LT_ cannot be operated as an electrode (*viz.* mono-tasking substrate) because of the insulating properties of PVA; (ii) pAuNP_PVA_HT_ shows the lowest sheet resistance (*i.e.* 0.23(±0.06) Ω per square). As mentioned, since PVA is a good insulator, the sintering step is mandatory for this application. On the other hand, the higher homogeneity of the sintered thin film achieved by PVA, allowed the best performance in terms of sheet resistance, highlighting the direct interplay between morphology and electronic features.

### Electrochemical-SERS characterization of the MBA functionalized thin film

**Fig. 4:**
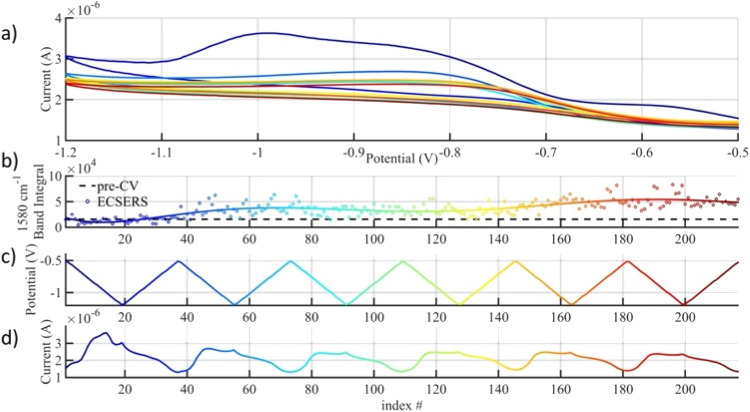
Electrochemical-SERS experiments run on an MBA functionalized pAuNP_PVA_HT_ electrode. (a) 6 cyclic voltammetry cycles were run between −0.5 and −1.2 V. (b) Raman spectra were simultaneously acquired during the voltammograms. The characteristic 1580 cm^−1^ band of MBA is recorded. The potential (c) and current (d) cycles are coherently shown with respect to panel b. The dashed line highlights the reference peak at 1580 cm^−1^ before electrochemical desorption as an internal benchmark.

So far, these nanostructured electrodes have been separately characterized by Raman spectroscopy and electrical tests (*i.e.* 4 probe measurement). For this reason, we decided to challenge our electrodes by using an electrochemical setup coupled with our SERS equipment (Fig. S17†). In partcular, the electrochemical desorption of MBA previously grafted onto our microelectrode (*viz.* pAuNP_PVA_HT_) has been performed. This proof-of-concept allows us to demonstrate the advantages of our multi-purpose electrodes. Consecutive voltammograms have been done in order to desorb electrochemically the MBA assembled onto the electrode. Three reductive peaks are recorded at −0.57 V, −0.8 V and −0.98 V, ascribed to Au(111), Au(100) and Au(110) facets, respectively (Fig. 4a).^56,57^ The reductive desorption is quantitative for the Au(111) facet after the first voltammogram, whereas the reductive desorption related to both Au(100) and Au(110) facets is gradual along the different voltammograms. This is caused by a partial MBA desorption ascribable to the geometry of our home-made sample holder together with any stirring of the electrolyte. Concerning the SERS spectra, the value of the MBA peak centred at 1580 cm^−1^ (Fig. 4b) has been recorded simultaneously with the voltammograms. A clear monotonic increment of the SERS intensity can be observed along the 6 CV cycles, with respect to the black dashed line in Fig. 4b, representing the average SERS intensity of the same band without any applied potential. As already mentioned, the sample holder cannot provide any stirring of the electrolyte. Counterintuitively, while the overall current is lowering along the CVs, the SERS signal is increasing. One should recall that far-field SERS signals are mostly ascribable to the molecules lying in the so-called *hot spots*.^58^ These are junctions in between Au nanoparticles in which local fields may accumulate, and the molecules are less favoured to diffuse toward the bulk solution.^59,60^ Consequently, the MBA molecules, lying on hot-spots, may guarantee a stable SERS signal. It is also reported that self-assembled monolayers (SAMs) of MBA on Au(111) facets foresee a tilted MBA orientation with respect to the Au surface.^61^ The desorption of the majority of the MBA molecules, as suggested by the current trend in Fig. 4d, may have the consequence of allowing a better re-orientation of the remaining molecules, according to the SERS surface selection rules.^62^ As suggested by the dipole derivative unit vector depicted in Fig. S18† for the MBA band at 1580 cm^−1^, the orientation of the molecules with respect to the plasmonic surface has a strong influence on the 1580 cm^−1^ peak intensity. The SERS results show, therefore, that the electrochemically driven molecular desorption favours rearrangements of the same, in which the molecular planes aligns gradually more vertical and perpendicular with respect to the metal surface.

## Conclusions

Two novel and inkjet printable formulations of AuNPs are presented. Both formulations meet the stringent requirements of green chemistry, because the NP synthesis has been achieved by laser ablation in an aqueous solution and the additives are well-known to be environment friendly. Our thin films have been deeply investigated in terms of morphological, optical and electrical properties. Our approach allows the manufacturing of large-scale nanotextured multi-purpose substrates, whose optoelectronic features show promising performances in terms of SERS signals and sheet resistances. Their performances are at the state-of-the-art about electrodes and SERS substrates (see Table S1†), but, to the best of our knowledge, no example of multi-tasking substrates has been presented yet. For this reason, our multitasking electrodes succeeded in supporting the electrochemical-SERS measurement, aiming at deeper molecular characterization studies such as the electrodesorption of MBA molecules. In conclusion, we can safely state that this approach fulfils the requirements of large-area and environment-friendly electronics, thereby paving the way toward multi-functional and miniaturized devices.

## Author contributions

LL accounts for project conceptualization and supervision. SR and LL executed the synthesis and functionalization of the gold nanoparticles and the Raman. LL, SC, SB executed and analysed the ECSERS measurements. SR, MB and LL optimized and executed the inkjet printing. SC performed darkfield microscopy. MB performed electrical characterization of the thin films. All authors contributed to the results' discussion and manuscript preparation.

## Conflicts of interest

There are no conflicts to declare.

## Supplementary Material

NA-005-D2NA00917J-s001

## References

[cit1] Ricci S., Casalini S., Parkula V., Selvaraj M., Saygin G. D., Greco P., Biscarini F., Mas-Torrent M. (2020). Biosens. Bioelectron..

[cit2] Lin L. K., Tsai J. T., DÃ-Az-Amaya S., Oduncu M. R., Zhang Y., Huang P. Y., Ostos C., Schmelzel J. P., Mohammadrahimi R., Xu P., Ulloa Gomez A. M., Shuvo S. N., Raghunathan N., Zhang X., Wei A., Bahr D., Peroulis D., Stanciu L. A. (2021). ACS Appl. Mater. Interfaces.

[cit3] Sansoni S., De Bastiani M., Aydin E., Ugur E., Isikgor F. H., Al-zahrani A., Lamberti F., Laquai F., Meneghetti M. (2020). Adv. Mater. Technol..

[cit4] Zhumagali S., Isikgor F. H., Maity P., Yin J., Ugur E., De Bastiani M., Subbiah A. S., Mirabelli A. J., Azmi R., Harrison G. T., Troughton J., Aydin E., Liu J., Allen T., Rehman A., Baran D., Mohammed O. F., De Wolf S. (2021). Adv. Energy Mater..

[cit5] Silvestri A., Criado A., Poletti F., Wang F., Fanjul-bolado P., González-garcía M. B., García-astrain C., Liz-marzán L. M., Feng X., Zanardi C., Prato M. (2021). Adv. Funct. Mater..

[cit6] Kosmala T., Baby A., Lunardon M., Perilli D., Liu H., Durante C., Di Valentin C., Agnoli S., Granozzi G. (2021). Nat. Catal..

[cit7] Iffelsberger C., Pumera M. (2021). J. Mater. Chem. A.

[cit8] Iffelsberger C., Wert S., Matysik F. M., Pumera M. (2021). ACS Appl. Mater. Interfaces.

[cit9] Zabihipour M., Lassnig R., Strandberg J., Berggren M., Fabiano S., Engquist I., Andersson Ersman P. (2020). npj Flexible Electron..

[cit10] Nayak L., Mohanty S., Nayak S. K., Ramadoss A. (2019). J. Mater. Chem. C.

[cit11] Cummins G., Desmulliez M. P. Y. (2012). Circuit World.

[cit12] Zhan Z., An J., Wei Y., Tran V. T., Du H. (2017). Nanoscale.

[cit13] Yang C. Y., Stoeckel M. A., Ruoko T. P., Wu H. Y., Liu X., Kolhe N. B., Wu Z., Puttisong Y., Musumeci C., Massetti M., Sun H., Xu K., Tu D., Chen W. M., Woo H. Y., Fahlman M., Jenekhe S. A., Berggren M., Fabiano S. (2021). Nat. Commun..

[cit14] Viola F. A., Barsotti J., Melloni F., Lanzani G., Kim Y. H., Mattoli V., Caironi M. (2021). Nat. Commun..

[cit15] Mu L., He M., Jiang C., Wang J., Mai C., Huang X., Zheng H., Wang J., Zhu X. H., Peng J. (2020). J. Mater. Chem. C.

[cit16] Yang P., Zhang L., Kang D. J., Strahl R., Kraus T. (2020). Adv. Opt. Mater..

[cit17] Schackmar F., Eggers H., Frericks M., Richards B. S., Lemmer U., Hernandez-Sosa G., Paetzold U. W. (2021). Adv. Mater. Technol..

[cit18] Eggenhuisen T. M., Galagan Y., Biezemans A. F. K. V., Slaats T. M. W. L., Voorthuijzen W. P., Kommeren S., Shanmugam S., Teunissen J. P., Hadipour A., Verhees W. J. H., Veenstra S. C., Coenen M. J. J., Gilot J., Andriessen R., Groen W. A. (2015). J. Mater. Chem. A.

[cit19] Pietsch M., Schlisske S., Held M., Strobel N., Wieczorek A., Hernandez-Sosa G. (2020). J. Mater. Chem. C.

[cit20] Trotter M., Juric D., Bagherian Z., Borst N., Glaser K., Meissner T., von Stetten F., Zimmermann A. (2020). Sensors.

[cit21] Minari T., Kanehara Y., Liu C., Sakamoto K., Yasuda T., Yaguchi A., Tsukada S., Kashizaki K., Kanehara M. (2014). Adv. Funct. Mater..

[cit22] Langer J., de Aberasturi D. J., Aizpurua J., Alvarez-Puebla R. A., Auguié B., Baumberg J. J., Bazan G. C., Bell S. E. J., Boisen A., Brolo A. G., Choo J., Cialla-May D., Deckert V., Fabris L., Faulds K., Javier García de Abajo F., Goodacre R., Graham D., Haes A. J., Haynes C. L., Huck C., Itoh T., Käll M., Kneipp J., Kotov N. A., Kuang H., Le Ru E. C., Lee H. K., Li J. F., Ling X. Y., Maier S. A., Mayerhöfer T., Moskovits M., Murakoshi K., Nam J. M., Nie S., Ozaki Y., Pastoriza-Santos I., Perez-Juste J., Popp J., Pucci A., Reich S., Ren B., Schatz G. C., Shegai T., Schlücker S., Tay L. L., George Thomas K., Tian Z. Q., van Duyne R. P., Vo-Dinh T., Wang Y., Willets K. A., Xu C., Xu H., Xu Y., Yamamoto Y. S., Zhao B., Liz-Marzán L. M. (2020). ACS Nano.

[cit23] Litti L., Reguera J., Garcıa de Abajo F. J., Meneghetti M., Liz-Marzán L. M. (2020). Nanoscale Horiz..

[cit24] Litti L., Colusso A., Pinto M., Ruli E., Scarsi A., Ventura L., Toffoli G., Colombatti M., Fracasso G., Meneghetti M. (2020). Sci. Rep..

[cit25] Litti L., Trivini S., Ferraro D., Reguera J. (2021). ACS Appl. Mater. Interfaces.

[cit26] Bell S. E. J., Charron G., Cortés E., Kneipp J., de la Chapelle M. L., Langer J., Procházka M., Tran V., Schlücker S. (2020). Angew. Chem., Int. Ed..

[cit27] Novara C., Petracca F., Virga A., Rivolo P., Ferrero S., Chiolerio A., Geobaldo F., Porro S., Giorgis F. (2014). Nanoscale Res. Lett..

[cit28] Godoy N. V., García-Lojo D., Sigoli F. A., Pérez-Juste J., Pastoriza-Santos I., Mazali I. O. (2020). Sens. Actuators, B.

[cit29] Yu W. W., White I. M. (2010). Anal. Chem..

[cit30] Miccichè C., Arrabito G., Amato F., Buscarino G., Agnello S., Pignataro B. (2018). Anal. Methods.

[cit31] Piotto V., Litti L., Meneghetti M. (2020). J. Phys. Chem. C.

[cit32] Fazio E., Gökce B., De Giacomo A., Meneghetti M., Compagnini G., Tommasini M., Waag F., Lucotti A., Zanchi C. G., Ossi P. M., Dell’aglio M., D’urso L., Condorelli M., Scardaci V., Biscaglia F., Litti L., Gobbo M., Gallo G., Santoro M., Trusso S., Neri F. (2020). Nanomaterials.

[cit33] Piotto V., Litti L., Omelyanchik A., Martucci A., Riello P., Peddis D., Meneghetti M. (2022). J. Mater. Chem. C.

[cit34] Sharma B., Rabinal M. K. (2015). J. Alloys Compd..

[cit35] Bergquist L., Hegmann T. (2017). ChemNanoMat.

[cit36] Abarghoei S., Fakhri N., Borghei Y. S., Hosseini M., Ganjali M. R. (2019). Spectrochim. Acta Mol. Biomol. Spectrosc..

[cit37] Liu Z., Lanier O. L., Chauhan A. (2020). Nanomaterials.

[cit38] Pimpang P., Choopun S. (2011). Chiang Mai J. Sci..

[cit39] Mekhmouken S., Battaglini N., Mattana G., Maurin A., Zrig S., Piro B., Capitao D., Noel V. (2021). Electrochem. Commun..

[cit40] Cui W., Lu W., Zhang Y., Lin G., Wei T., Jiang L. (2010). Colloids Surf., A.

[cit41] Carvajal S., Fera S. N., Jones A. L., Baldo T. A., Mosa I. M., Rusling J. F., Krause C. E. (2018). Biosens. Bioelectron..

[cit42] Thomas P. S., Guerbois J. P., Russell G. F., Briscoe B. J. (2001). J. Therm. Anal. Calorim..

[cit43] Basik M., Mobin M., Shoeb M. (2020). Sci. Rep..

[cit44] David Avnir L. E., Jin H., Armstrong J., Cong P., Menagen B., Igaher L., Beale A. M. (2020). Angew. Chem., Int. Ed..

[cit45] Lamont C. A., Eggenhuisen T. M., Coenen M. J. J., Slaats T. W. L., Andriessen R., Groen P. (2015). Org. Electron..

[cit46] Hoath S. D., Harlen O. G., Hutchings I. M. (2012). J. Rheol..

[cit47] Begines B., Alcudia A., Aguilera-Velazquez R., Martinez G., He Y., Wildman R., Sayagues M. J., Jimenez-Ruiz A., Prado-Gotor R. (2019). Sci. Rep..

[cit48] Jing C., Fang Y. (2007). Chem. Phys..

[cit49] Zubair N. A., Rahman N. A., Lim H. N., Zawawi R. M., Sulaiman Y. (2016). RSC Adv..

[cit50] Wu D., Li J., Ren B., Tian Z. (2008). Chem. Soc. Rev..

[cit51] Lenzi E., Litti L., de Aberasturi D. J., Henriksen-Lacey M., Liz-Marzán L. M. (2020). J. Raman Spectrosc..

[cit52] Le Ru E. C., Blackie E., Meyer M., Etchegoin P. G. (2007). J. Phys. Chem. C.

[cit53] Bell S. E. J., Charron G., Cortés E., Kneipp J., Lamy de la Chapelle M., Chapelle D., Langer J., Prochazka M., Tran V., Schlucker S. (2020). Angew. Chem., Int. Ed..

[cit54] Venugopal A., Pirkle A., Wallace R. M., Colombo L., Vogel E. M. (2009). AIP Conf. Proc..

[cit55] Keyes D. B. (1928). J. Chem. Educ..

[cit56] Arihara K., Ariga T., Takashima N., Arihara K., Okajima T., Kitamura F., Tokuda K., Ohsaka T. (2003). Phys. Chem. Chem. Phys..

[cit57] González-Rubio G., Scarabelli L., Guerrero-Martínez A., Liz-Marzán L. M. (2020). ChemNanoMat.

[cit58] Litti L., Meneghetti M. (2019). Phys. Chem. Chem. Phys..

[cit59] Novčić K. A., Iffelsberger C., Pumera M. (2022). J. Mater. Chem. A.

[cit60] Iffelsberger C., Ng S., Martin P. (2022). Chem. Eng. J..

[cit61] Pensa E., Rubert A. A., Benitez G., Carro P., Orive A. G., Creus A. H., Salvarezza R. C., Vericat C. (2012). J. Phys. Chem. C.

[cit62] Le Ru E. C. L., Meyer S. A., Artur C., Etchegoin P. G., Grand J., Lang P., Maurel F. (2011). Chem. Commun..

