# Supplementary material for: High performance multi-purpose nanostructured thin films by inkjet printing: Au micro-electrodes and SERS substrates
Source: Nanoscale Adv. 2023 Feb 16;5(7):1970–7. doi: 10.1039/d2na00917j (PMC10044483; doi:10.1039/d2na00917j)
Supplement: NA-005-D2NA00917J-s001 [file NA-005-D2NA00917J-s001.pdf]

Electronic Supplementary Material (ESI) for Nanoscale Advances.
This journal is © The Royal Society of Chemistry 2023

Supporting information

High performance multi-purpose nanostructured thin films by inkjet printing: Au micro-electrodes and SERS substrates

Simona Ricci,[a] Marco Buonomo,[b] Stefano Casalini,[a] Sara Bonacchi,[a] Moreno Meneghetti,[a] Lucio Litti[a,*]

[a]Department of Chemical Sciences, University of Padova, Via Marzolo, 1, 35131, Padova, Italy.

* lucio.litti@unipd.it

[b]Department of Informatic Engineering, University of Padova, Via Gradenigo 6/b 35131, Padova, Italy

MATERIALS AND INSTRUMENT: Sodium chloride, hydrochloric acid (37% wt), polyvinyl alcohol (22000 MW) were purchased from Fluka, sulfuric acid (95-97% wt), nitric acid (65% wt), isopropanol, L-cysteine (non-animal origin) and p-mercaptobenzoic acid (4-MBA) were purchased from Sigma-Aldrich. Hydrogen peroxide (30% wt) was obtained from Merck, microscope glass slides (1 mm thick) were provided from Thermo Fisher Scientific, sodium hydroxide was procured from Carlo Erba. FEI Tecnai G2 12 operating at 100 kV and equipped with a TVIPS CCD camera was used for transmission electron microscopy. The Agilent Cary 5000 UV-vis-NIR spectrometer was used for the optical extinction characterization. SEM was performed with a Zeiss Sigma HD microscope, equipped with a Schottky FEG source, one detector for backscattered electrons and two detectors for secondary electrons (InLens and Everhart Thornley). The microscope is coupled to an EDX detector (from Oxford Instruments, x-act PentaFET Precision) for X-rays microanalysis, working in energy-dispersive mode. Topographic analysis was performed using an Agilent 5500 SPM atomic force microscope (AFM) in intermittent contact mode equipped with a Mikromasch HQ: XSC11 / AL BS probe characterized by a tip of radius of curvature <10 nm, mean force constant 42 N / m and frequency 350 kHz. The images were acquired at ambient temperature and data were processed using WSxM software.[1] Raman measurements have been performed with a Renishaw InVia μRaman equipped with a He-

Ne laser emitting at 632.8 nm (nominal power of 15mW) and a diode laser emitting at 785 nm (nominal power of 30mW). Sheet resistance has been measured with a four-probe system from Ossila, whose probe spacing is equal to 1.27 mm; three runs were recorded and mediated for each sample. Electrochemical measures were recorded using a CHI 660c Electrochemical work station.

LASER ABLATION: Gold nanoparticles were prepared by laser ablation synthesis in solution (LASiS) as previously reported.[2] In brief, a plate of pure gold was placed in the bottom of a glass flask filled with 10µM NaCl water solution. A 9ns pulsed Nd:YAG laser (Quantel QSmart 850), operating at the 1064 nm fundamental wavelength and at 20Hz repetition rate, was focused on the target surface. The ablation was periodically monitored recording extinction spectra of the colloid and stopped once the AuNP concentration reached about 1 to 2 nM concentration. The nanoparticles concentration was calculated according to their molar extinction coefficient, obtained by the model presented in reference.[3]

AuNP_Cys: a fairly assumed sub-monolayer coverage of L-cysteine on AuNP was obtained by adding a stock solution of L-cysteine to the AuNP solution prepared by LASiS (2.0 nM) reaching a final concentration of L-Cys equal to 0.05 µM, so that about 24 molecules of L-cysteine per AuNP. The pH was adjusted to 10 by adding some drops of NaOH 1 M. The solution was stirred overnight at room temperature. Afterwards, centrifugation at 3000 rcf for 30 minutes was carried out. The supernatant was removed achieving a final concentration of AuNPs equal to $1.6\cdot10^{-8}$ M. This ink formulation was used without further processing.

AuNP_PVA: 10 mg/mL PVA in water was added to the AuNP solution prepared by LASiS (1.2 nM), achieving a final concentration of PVA equal to 1 mg/mL. The colloid was centrifuged at 3000 rcf for 30 minutes. The supernatant was removed. The obtained concentration of AuNPs was equal to $2.4\cdot10^{-8}$ M. This ink formulation was used without further processing.

Electrochemical-SERS measures: a brand-new electrode was inkjet-printed following the procedure already described for the pAuNP_PVA_{HT} series, but designed as in Figure S15a. It was incubated overnight in MBA 2.6mM in NaOH 0.1M and rinsed with NaOH 0.1M before the experiments. A dedicated electrochemical cell was 3D-printed as well (Figure S15b), in which the electrode may be placed at the bottom and crewed in between the cell and a steal plate. An Ag/AgCl reference electrode and a Pt wire, as counter electrode, were

used. The potentiometer was set to run multiple cycles in between -0.5 and -1.2V, at a scan rate or 0.01V/s. NaOH 0.1M was used as electrolyte. All of these were placed on the sample plate of the Raman instrument and continuous spectra acquisition (633 nm laser excitation at 6mW, 3s acquisition time, 10x objective magnification) were run along the cyclic voltammetry measurements. Raman spectra were baseline corrected and the band at about 1580 cm^{-1} was integrated for further analysis.

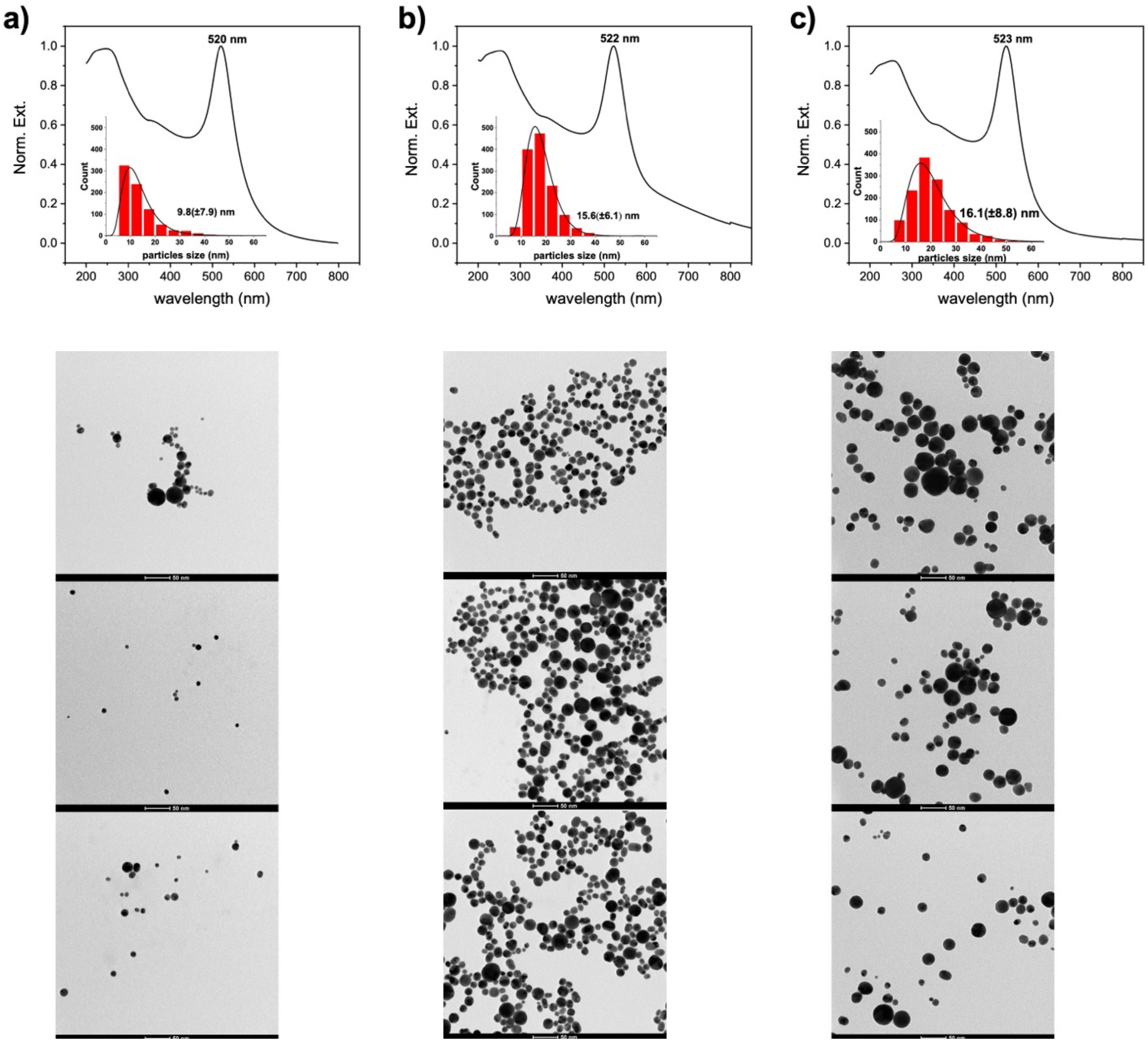

Figure S1. UV-vis spectra, TEM images and nanoparticles size distribution of a) AuNPs prepared by laser ablation, b) AuNP_Cys and c) AuNP_PVA inks. The redshifts observed upon ink formulation are in agreement with the surface plasmon shift due to the particles coverage with cysteine and PVA that induce an increase of the local environmental refractive index.[4]

In **Figure. S1** the UV-vis spectra of the pristine AuNPs, AuNP_Cys and AuNP_PVA are reported together with their TEM images and their corresponding size distributions. From the UV-vis spectra, it is possible to locate the plasmonic peak of the AuNPs at 520 nm for the as-synthesised AuNP, and at 522 nm and 523 nm for the AuNP_Cys and AuNP_PVA inks respectively. TEM analysis reveals a slightly higher and less polydisperse distribution, at 15.6(±6.1) nm and 16.1(±8.8) nm for AuNP_Cys and AuNP_PVA, respectively, compared to the laser ablated AuNPs spheres, which have a diameters distribution centred at 9.8(±7.9) nm. This dimension difference is a consequence of the sorting obtained by centrifugation, which is well-known to separate particles by size, with larger particles sedimenting faster than the smaller ones, indeed providing the narrower size distribution.

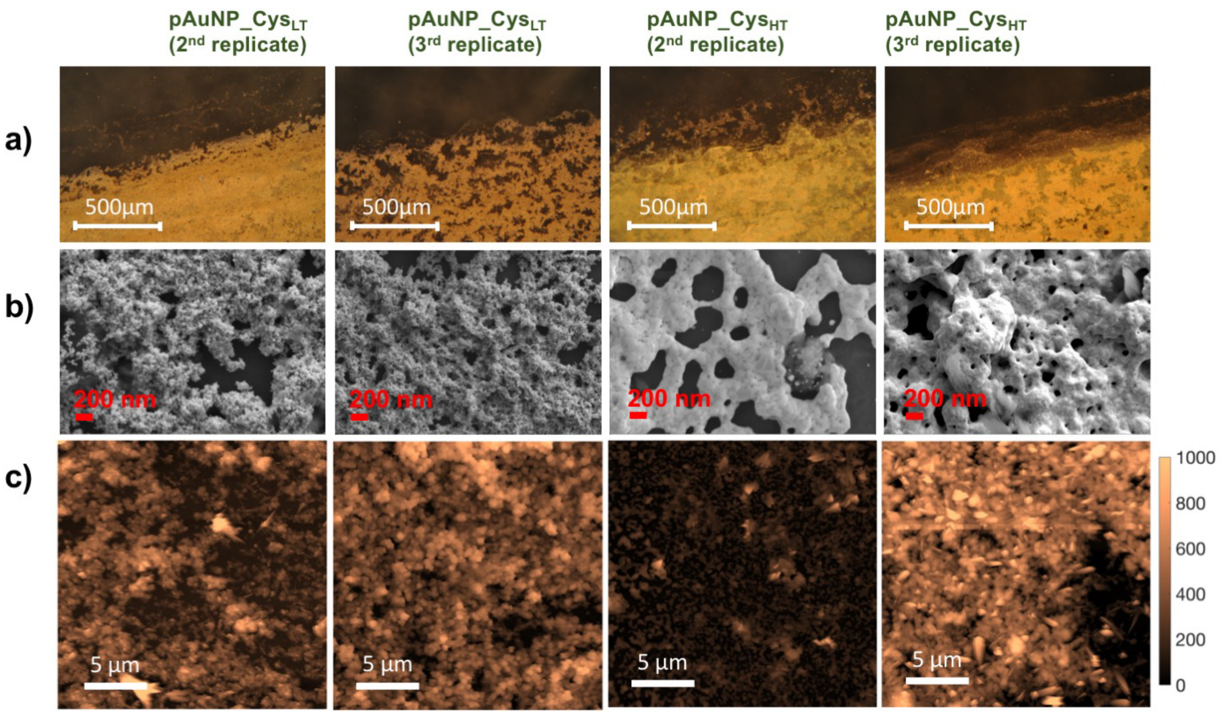

Figure S2. Morphological characterization of the different substrates: a) dark-field optical microscopy images with a 5x objective, b) SEM images, c) AFM topography images, the colormap represents the thickness in nanometers and is coherent for all the AFM images.

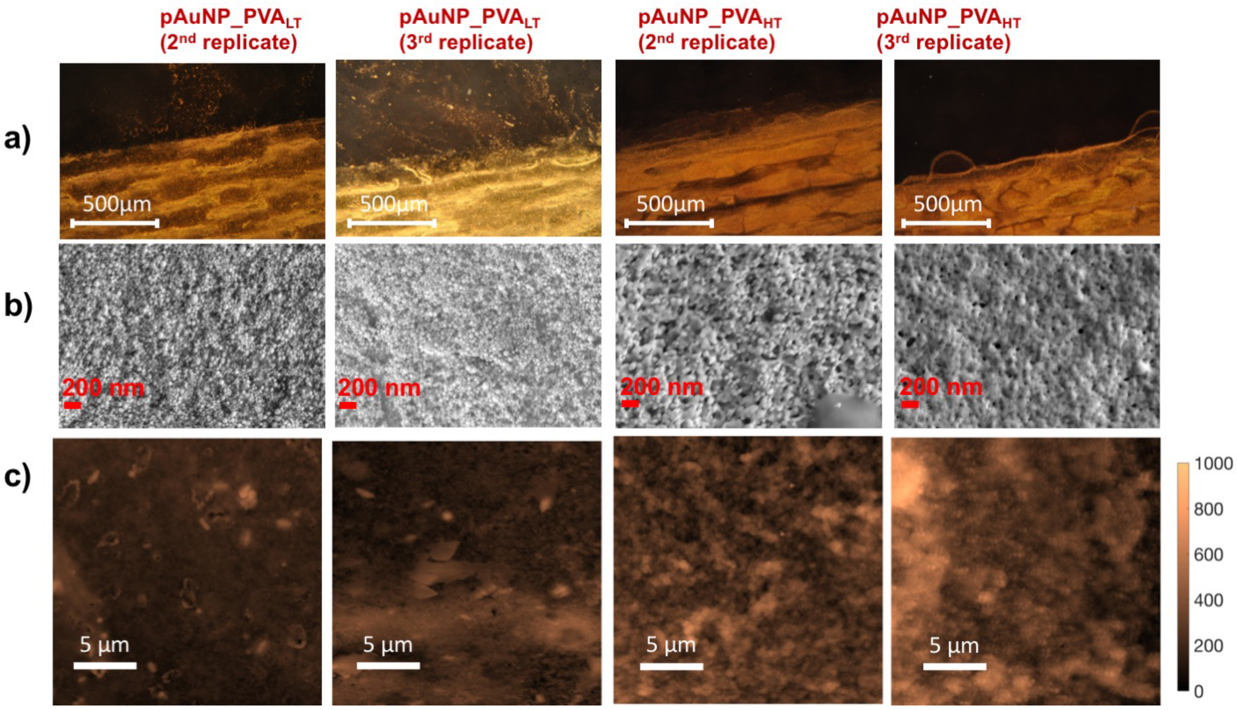

Figure S3. Morphological characterization of the different substrates: a) dark-field optical microscopy images with a 5x objective, b) SEM images, c) AFM topography images, the colormap represents the thickness in nanometers and is coherent for all the AFM images.

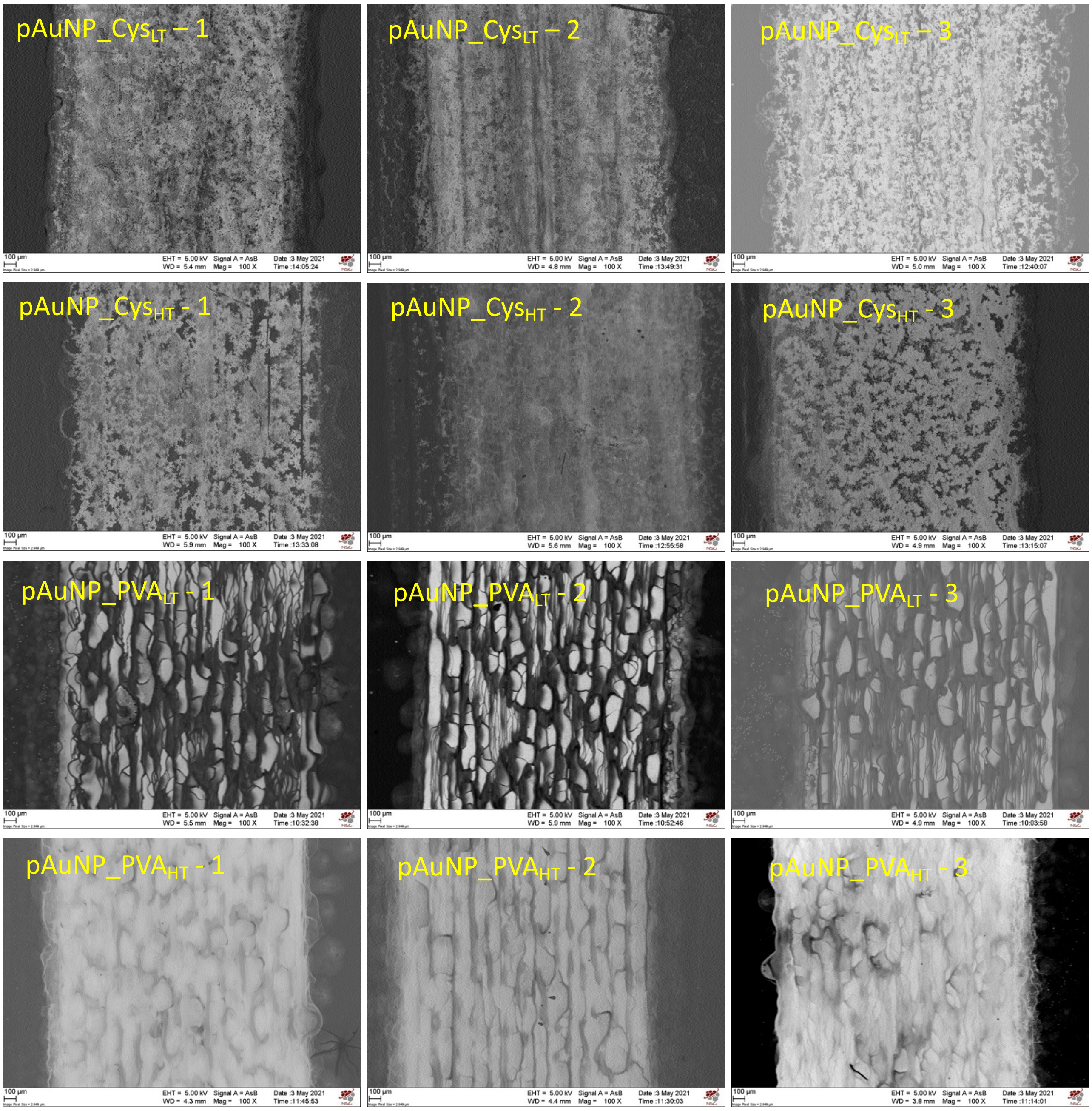

Figure S4. SEM images at 100x magnification

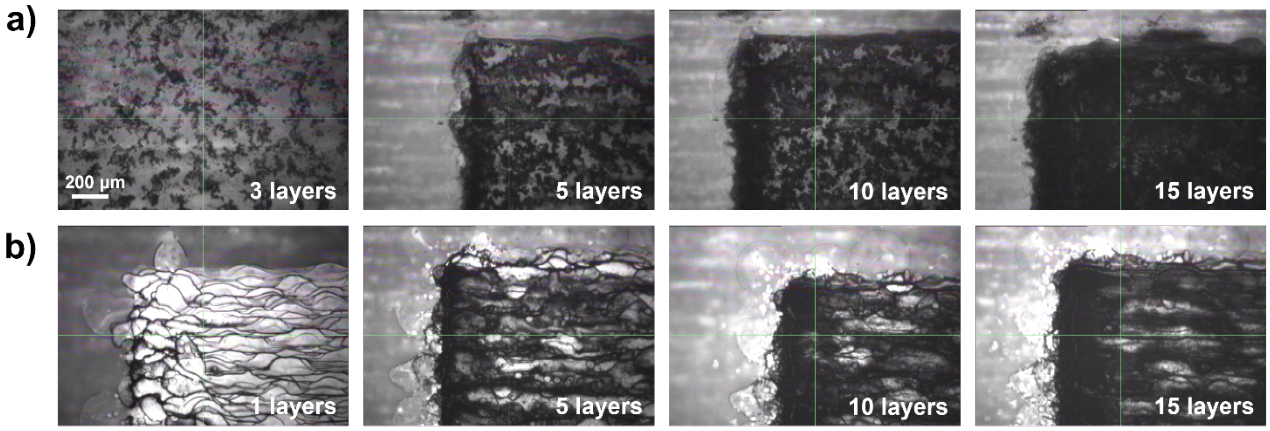

Figure S5. Fiducial camera images of the a) (AuNP_Cys)-based printing and b) (AuNP_PVA)-based printing taken at different stages of the printing procedure. Noteworthy, the AuNP_PVA ink ensures a complete surface coverage already after a single layer, whereas the AuNP_Cys ink shows a bunch-like deposition.

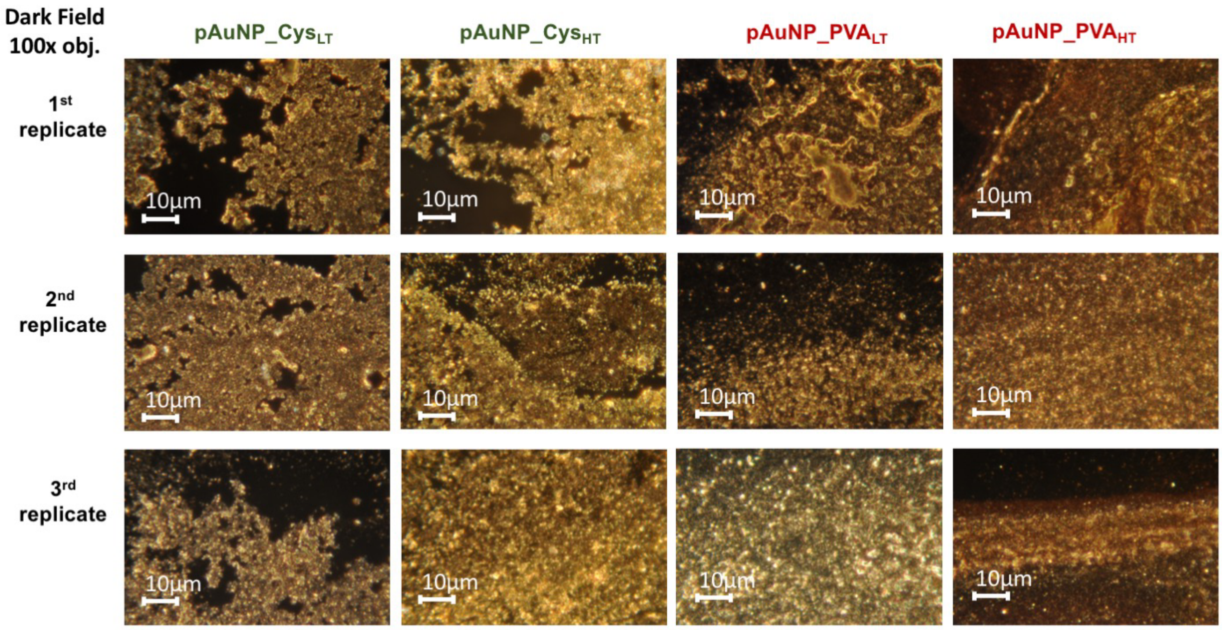

Figure S6: Morphological characterization of the different substrates using dark-field optical microscopy with a 100x objective.

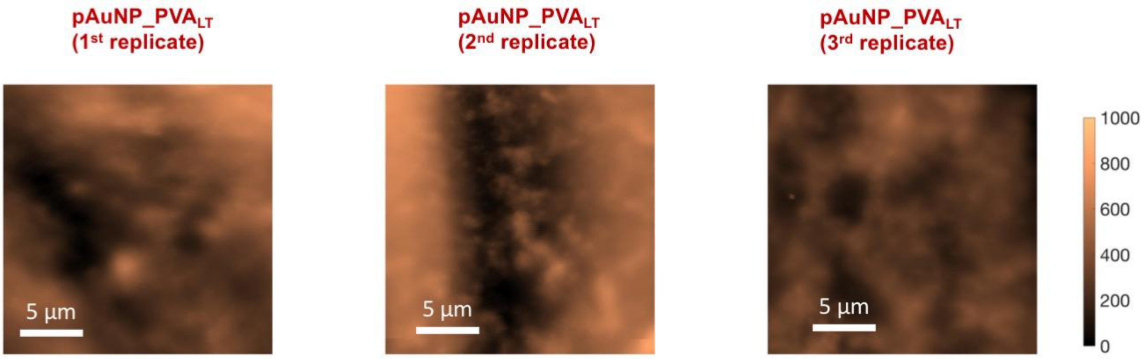

Figure S7: AFM topography images for the PVA rich regions in pAuNP_PVA$_{LT}$ samples, the colourmap represents the thickness in nanometers and is coherent for all the AFM images.

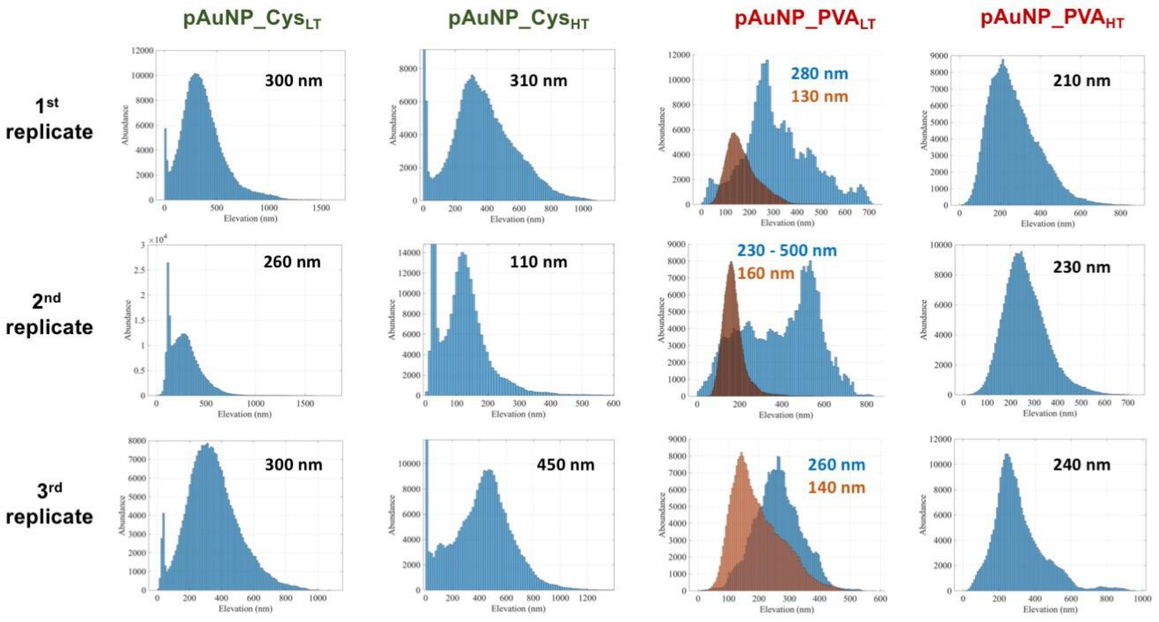

Figure S8: Distribution of the elevations measured for the AFM images of Figure 2 and S2, S3 and S5 (orange histograms).

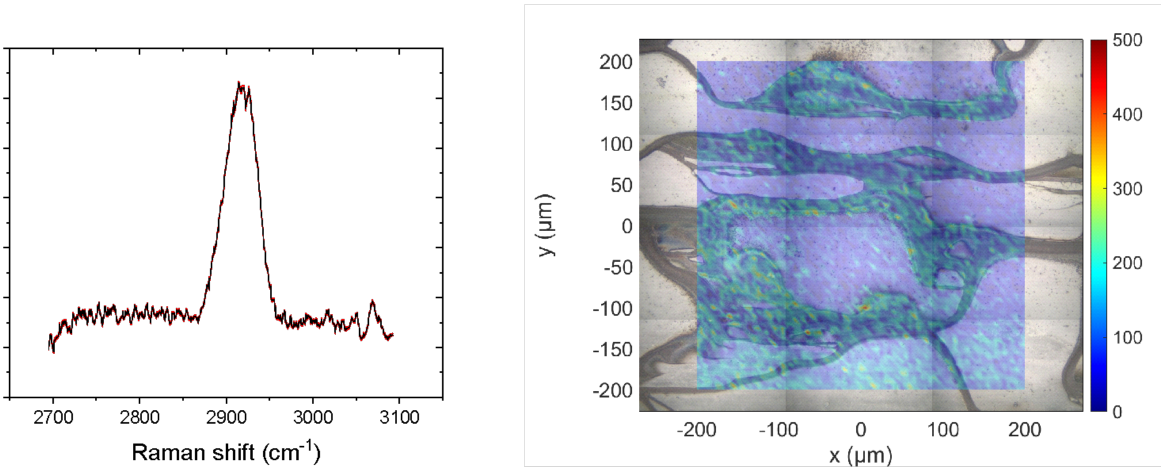

Figure S9. (a) Raman SERS spectrum of pAuNP_PVA_{LT} mediated over 6400 spectra collected in a portion of surface equal to 400x400 μm^2, along with the standard error of the mean. (b) μ-Raman map in which the colour gradient is referred to the intensity of the peak at 2915 cm-1, relative to the CH stretching of the PVA. It is apparent how the signal intensity is higher in the darker regions, confirming the concentration of the polymer in some areas.

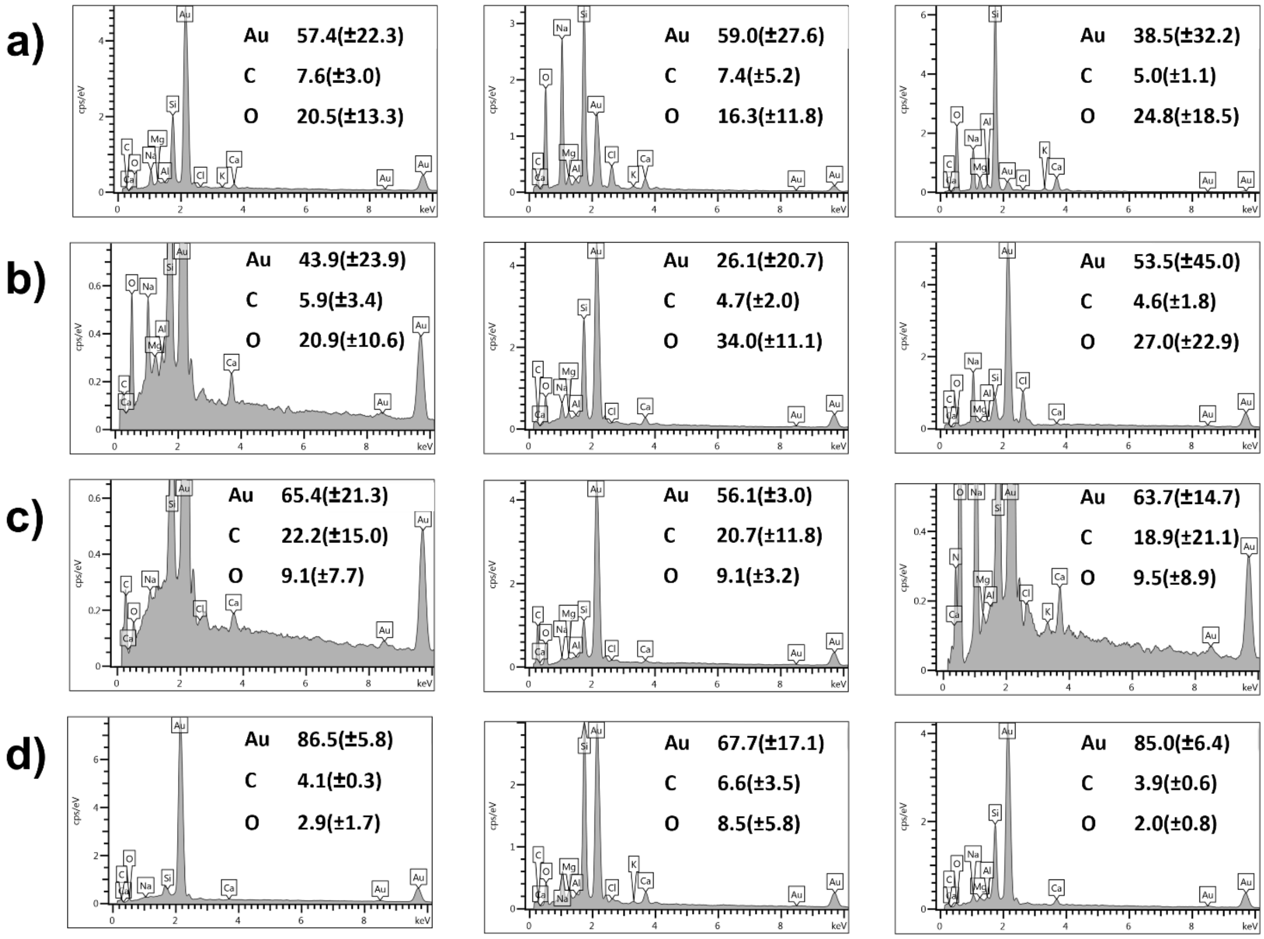

Figure S10. EDX results for a) pAuNP_Cys$_{LT}$, b) pAuNP_Cys$_{HT}$, c) pAuNP_PVA$_{LT}$ and d) pAuNP_PVA$_{HT}$. For each spectra the relative amount of Au, C and O are reported. The error is relative to at least three measurements in different points of the same sample.

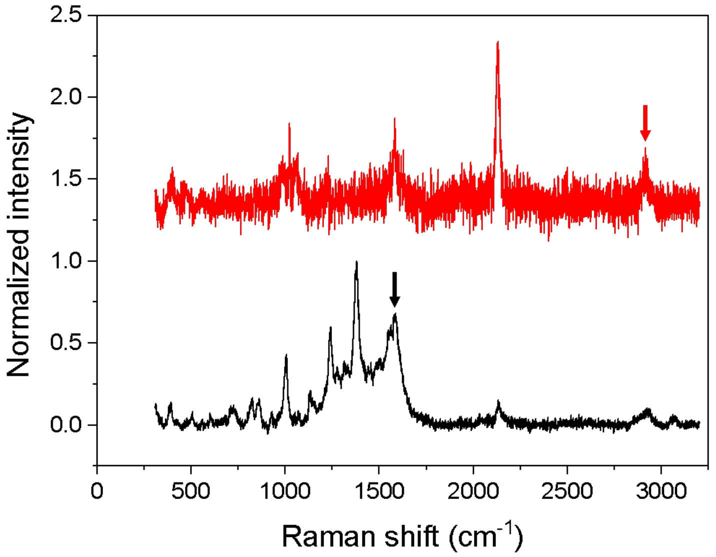

Figure S11. Raman spectra of the AuNP_cys ink (black curve) and AuNP_PVA ink (red curve). The spectra were acquired with the 633 nm excitation source using a 50x objective.

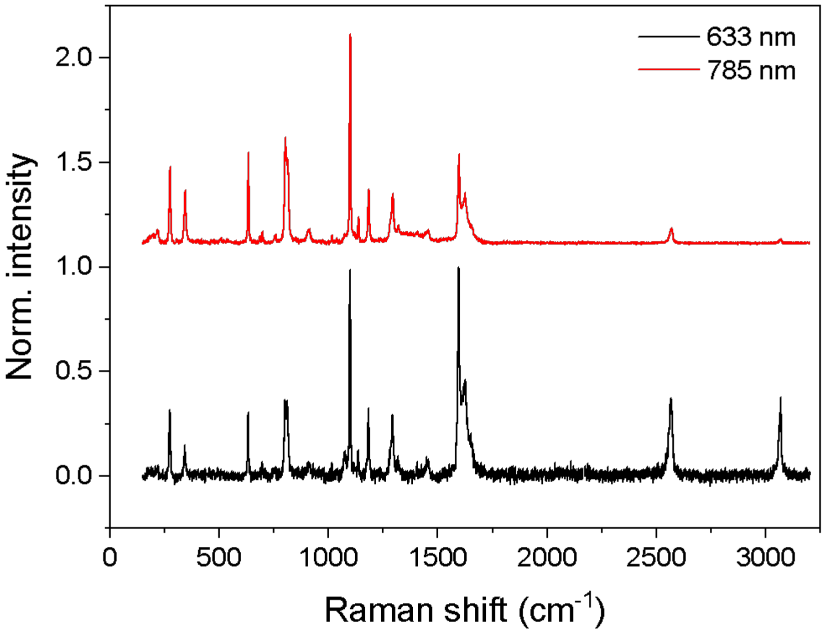

Figure S12: Raman spectra of the MBA powder acquired using a 50x objective and 633 nm excitation source (black curve) and 785 nm excitation source (red curve).

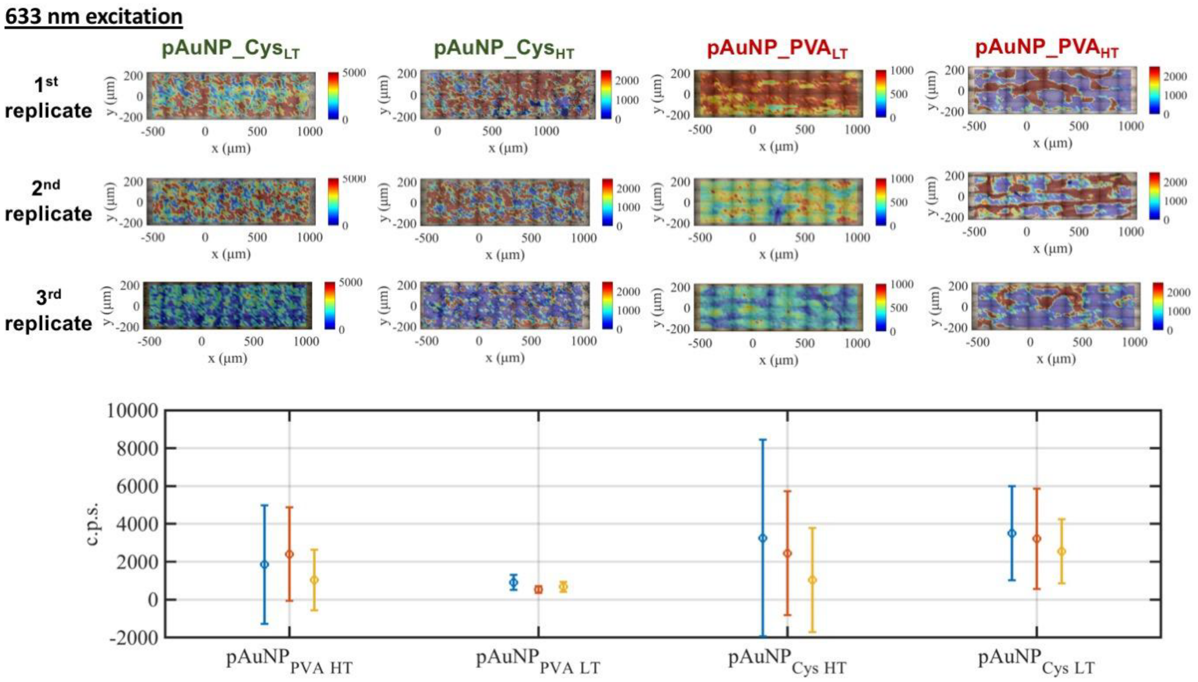

Figure S13: SERS maps relative to the inkjet-printed substrates functionalized with MBA. The false colours are relative to the intensities of the 1585 cm^{-1} MBA band. The plot at the bottom resumes the same band for all the samples in term of mean and standard deviation.

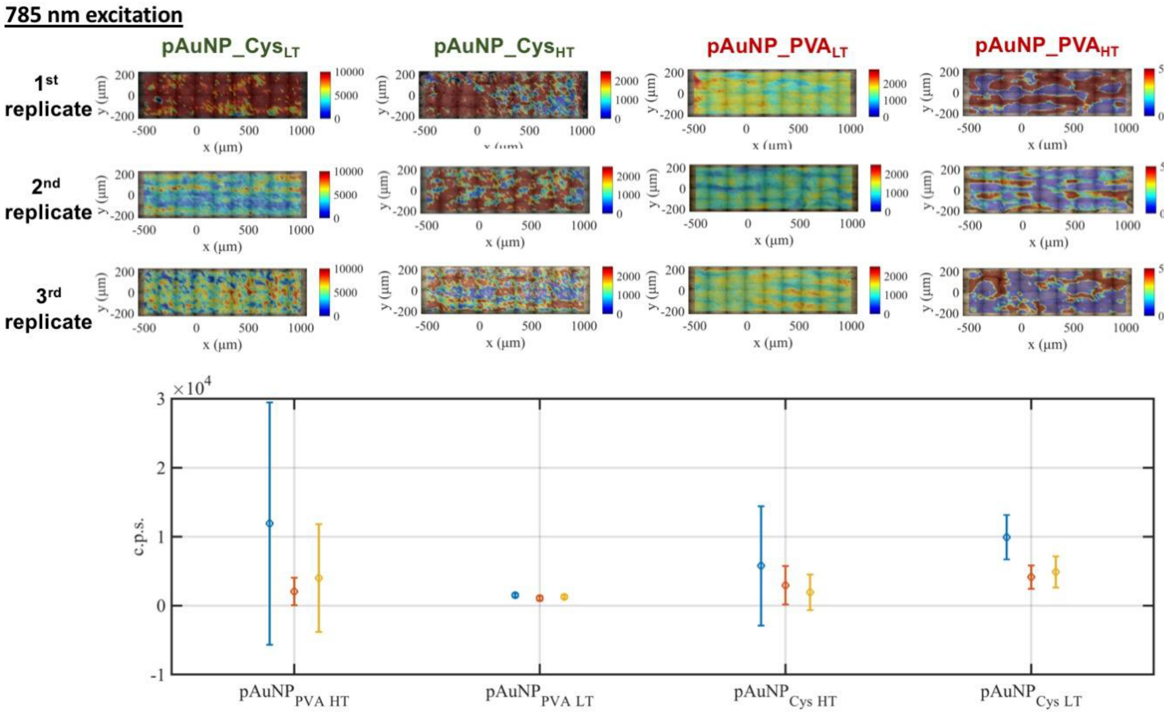

Figure S14: SERS maps relative to the inkjet-printed substrates functionalized with MBA. The false colours are relative to the intensities of the 1585 cm^{-1} MBA band. The plot at the bottom resumes the same band for all the samples in terms of mean and standard deviation.

PRINTING FEATURES: The electrodes were patterned on glass substrates employing the Dimatix Materials Printer (DMP-2800, Fujifilm) equipped with 10 pL droplet cartridges. The glass slides were cleaned by immersion in acid piranha for 30 minutes. Afterwards, they were abundantly rinsed with distilled water and dried by N_2 flow. The temperature of the platen was set to 40°C and the cartridge was let at ambient temperature. The waveform was optimized and it is illustrated in **Figure S14**. The firing voltage was adjusted in the range of 20-25 V, depending on the nozzle used. 10 µm of drop spacing was used for a total of 15 layers unless differently specified.

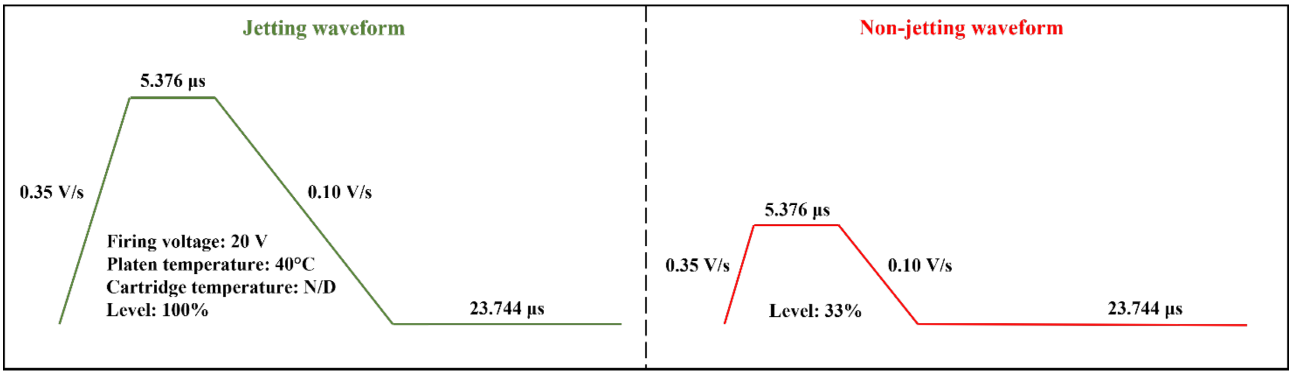

Figure S15: Waveform used and corresponding parameters for the jetting and non-jetting profile.

Table1. State-of-the-art inkjet printing SERS-active surfaces (yellow) and metal electrodes (green).

Materials	Substrate	Enhancement factor	Electrical features	Curing temperature	Laser	Application	Ref.
AuNP	Paper	Not reported	N/D	none	785 nm	SERS for sensing	5
AuNP	Paper	$2x10^6$ $1.4x10^6$	N/D	none	633 and 785 nm	SERS for sensing	6
AuNP	Paper	Not reported	N/D	none	632 nm	SERS for sensing	7
AgNP	Paper	$2x10^5$	N/D	none	633 nm	SERS for sensing	8
AgNP	Mesoporous silicon	10^8 (EARE)	N/D	none	514.5 nm	SERS for sensing	9
AgNP	Paper	Not reported	N/D	none	532 and 633 nm	SERS for sensing	10
AgNP	Glass	$>10^4$	N/D	none	633 nm	SERS for sensing	11
AuNP	Paper	N/D	0.06 Ω/cm	100°C (Interval times of 30 min)	Not used	Electrochemical sensor	12
Commercial silver ink and gold ink	Kapton	N/D	Not reported	330-400°C (silver ink) 250-330°C (gold ink)	Not used	Biosensing and OTFT	13
Commercial gold ink	ITO	N/D	2-100 Ω/sq	60°C 24h in vacuum	Not used	Electrochromic display	14
Commercial gold and silver nanoparticle ink	cyclic olefine copolymer (COC)	N/D	23 μΩ cm (resistivity)	130°C 1h + photonic curing	Not used	Sensing	15
AuNP	Kapton	N/D	0.22 Ω/sq	220°C 1h (each layer)	Not used	Bio-electrochemistry applications	16
AuNP	Plastics and paper	N/D	9 μΩ cm (resistivity)	R.T.	Not used	OTFT	17

ENHANCEMENT FACTOR CALCULATION PROCEDURE

A sample of MBA powder was used for the Raman measure (Figure S11), whereas for SERS experiment, a drop of 4-MBA solution (about 1 mM) was cast over the substrates and left to react for three hours without letting the drop dry. Afterwards, they were rinsed with water and dried gently under N_2 flow. At this point, a μ-Raman map was carried out to investigate the efficiency of the samples as SERS substrates.

For calculating the EF the following equation has been followed:

$$EF = \frac{I_{SERS}}{I_{bulk}} \frac{N_{bulk}}{N_{SERS}} \qquad (1)$$

where I_{SERS} is the surface-enhanced signal intensity of the probe at a specific band, I_{bulk} is the corresponding Raman intensity of its powder for the same band, N_{SERS} and N_{bulk} are the numbers of molecules probed, respectively for the SERS and Raman measurements.[18]

I_{bulk} and I_{SERS} were extrapolated from the experimental Raman and SERS spectra, respectively.

To determine N_{bulk}, an accurate approach was followedIt was assumed that the entire focal volume contributes to the measured signal. First, it is necessary to measure the effective sample volume irradiated by the laser. The confocal volume is given by:[19]

$$V_{confocal} = \pi^{\frac{3}{2}} \cdot w_0^2 \cdot z_0 \qquad (2)$$

where w_0 is the probe volume radius and z_0 is the half-height. Z_0 can be measured experimentally and w_0 can be derived by the Rayleigh limit given by:

$$w_0 = \sqrt{\frac{z_0 \cdot \lambda}{\pi \cdot n}} \qquad (3)$$

where n is the refractive index of the sample.

Z_0 was experimentally measured by acquiring Raman spectra along the z-direction. From a gaussian fit of the curve obtained by plotting the intensity of the 4-MBA powder signal *vs* the position along the z-direction, the full width at half maximum, FWHM, was extrapolated. It is known that this value corresponds to $2z_0$.[19]

It is also known that the density of the 4-MBA is equal to 1.5 $g \cdot cm^{-3}$, and thus, the number of molecules of 4-MBA probed by the Raman measure on the powder sample was accordingly calculated.[20]

A different approach was used for the calculation of N_{SERS}. In this case, not the confocal volume was used, but the sample surface under the laser spot. Again, z_0 was measured experimentally by acquiring Raman spectra of the printed sample along the z-direction and, consequently, w_0 was calculated. The area of the laser spot (*A*) was then given by:

$$A = \pi \cdot w_0^2 \qquad (4)$$

Ideally, it has been considered that a monolayer of 4-MBA was formed on top of the printed sample. The geometric area under laser irradiation does not rigorously correspond to the real area of the sample accessible to the 4-MBA molecules, due to the roughness and nanostructured surfaces of the samples. Thus, to have an estimation as reliable as possible, the roughness measured by AFM measurements were employed. From the AFM, the real surface area was calculated, and thus, from the surface hindrance of the 4-MBA molecules, which is known to be equal to 0.38 nm^2,[21] N_{SERS} was estimated.

The measurements and relative calculations were performed for both the 633 and 785 nm excitations.

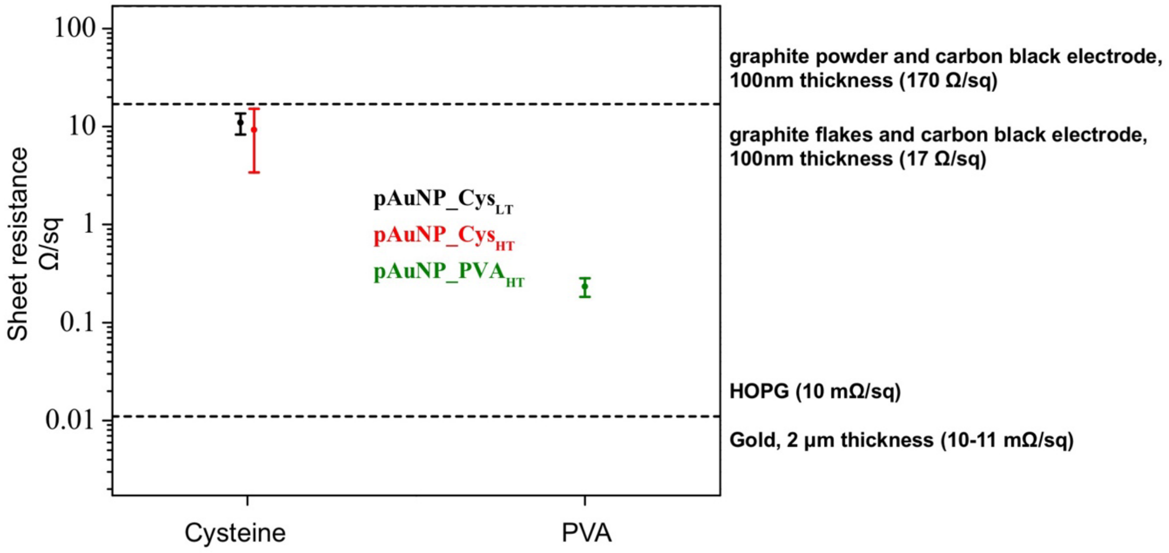

Figure S16: Sheet resistances of pAuNP_CysLT (in black), pAuNP_CysLT (in red) and pAuNP_CysLT (in green). Three replicates of each sample are averaged. The dashed line represents the sheet resistance of different electrodes reported in literature (Ref 22,23)

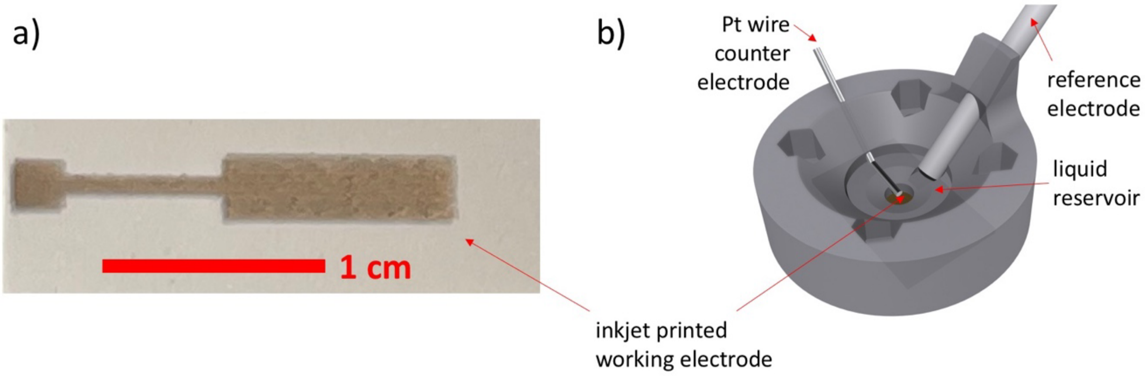

Figure S17: a) The pAuNP_PVA$_{HT}$ electrode printed for the Electrochemical-SERS experiments, so to make it fit at the bottom of the 3D-printed cell. b) representation of the 3D-printed electrochemical cell designed to guest the inkjet-printed electrode of (a) at its bottom.

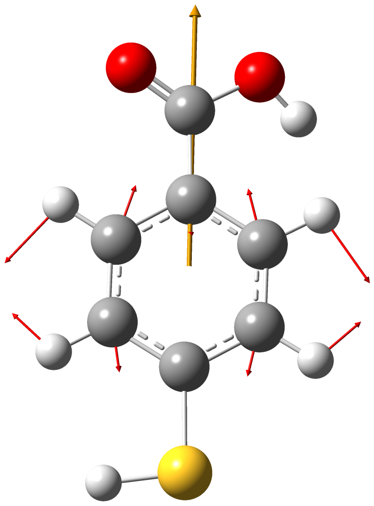

Figure S18: DFT calculation (b3lyp/6-311+G(d,p) functional and basis set) for the 1580cm^{-1} Raman peak of MBA, where the displacement vectors are represented in red and the dipole derivative unit vector in orange.

REFERENCES

1 A. You, M. A. Y. Be and I. In, *Rev. Sci. Instrum.*, 2007, **78**, 013705.

2 V. Piotto, L. Litti and M. Meneghetti, *J. Phys. Chem. C*, 2020, **124**, 4820.

3 L. Litti and M. Meneghetti, *Phys. Chem. Chem. Phys.*, 2019, **21**, 15515–15522.

4 M. Condorelli, L. Litti, M. Pulvirenti, V. Scardaci, M. Meneghetti and G. Compagnini, *Appl. Surf. Sci.*, 2021, **566**, 150701.

5 E. P. Hoppmann, W. W. Yu and I. M. White, *Methods*, 2013, **63**, 219–224.

6 N. V. Godoy, D. García-Lojo, F. A. Sigoli, J. Pérez-Juste, I. Pastoriza-Santos and I. O. Mazali, *Sensors Actuators, B Chem.*, 2020, **320**, 128412.

7 W. J. Liao, P. K. Roy and S. Chattopadhyay, *RSC Adv.*, 2014, **4**, 40487–40493.

8 W. W. Yu and I. M. White, *Anal. Chem.*, 2010, **82**, 9626.

9 C. Novara, F. Petracca, A. Virga, P. Rivolo, S. Ferrero, A. Chiolerio, F. Geobaldo, S. Porro and F. Giorgis, *Nanoscale Res. Lett.*, 2014, **9**, 527.

10 N. C. T. Martins, S. Fateixa, T. Fernandes, H. I. S. Nogueira and T. Trindade, *ACS Appl. Nano Mater.*, 2021, **4**, 4484–4495.

11 C. Miccichè, G. Arrabito, F. Amato, G. Buscarino, S. Agnello and B. Pignataro, *Anal. Methods*, 2018, **10**, 3215–3223.

12 T. Kant, K. Shrivas, K. Tapadia, R. Devi, V. Ganesan and M. K. Deb, *New J. Chem.*, 2021, **45**, 8297–8305.

13 L. K. Lin, J. T. Tsai, S. DÃ-Az-Amaya, M. R. Oduncu, Y. Zhang, P. Y. Huang, C. Ostos, J.

P. Schmelzel, R. Mohammadrahimi, P. Xu, A. M. Ulloa Gomez, S. N. Shuvo, N. Raghunathan, X. Zhang, A. Wei, D. Bahr, D. Peroulis and L. A. Stanciu, *ACS Appl. Mater. Interfaces*, 2021, **13**, 11369–11384.

14 M. Pietsch, S. Schlisske, M. Held, N. Strobel, A. Wieczorek and G. Hernandez-Sosa, *J. Mater. Chem. C*, 2020, **8**, 16716.

15 M. Trotter, D. Juric, Z. Bagherian, N. Borst, K. Glaser, T. Meissner, F. von Stetten and A. Zimmermann, *Sensors*, 2020, **20**, 1333.

16 S. Mekhmouken, N. Battaglini, G. Mattana, A. Maurin, S. Zrig, B. Piro, D. Capitao and V. Noel, *Electrochem. commun.*, 2021, **123**, 106918.

17 T. Minari, Y. Kanehara, C. Liu, K. Sakamoto, T. Yasuda, A. Yaguchi, S. Tsukada, K. Kashizaki and M. Kanehara, *Adv. Funct. Mater.*, 2014, **24**, 4886.

18 E. C. Le Ru, E. Blackie, M. Meyer and P. G. Etchegoin, *J. Phys. Chem. C*, 2007, **111**, 13794–13803.

19 J. R. Lakowicz, *Principles of fluorescence spectroscopy*, 2006.

20 P. Ji, Z. Mao, Z. Wang, X. Xue, Y. Zhang, J. Lv and X. Shi, *Nanomaterials*, 2019, **9**, 983.

21 I. Ragheb, M. Braïk, S. Lau-truong, A. Belkhir, G. Lévi, J. Aubard, L. Boubekeur-lecaque and N. Félidj, *Nanomaterials*, 2020, **10**, 2201.

22 A. Venugopal, A. Pirkle, R. M. Wallace, L. Colombo and E. M. Vogel, *AIP Conf. Proc.*, 2009, **1173**, 324–327.

23 D. B. Keyes, *J. Chem. Educ.*, 1928, **5**, 241.
